# Development and benchmark to obtain AMBER parameters dataset for non-standard amino acids modified with 4-hydroxy-2-nonenal

**DOI:** 10.1016/j.dib.2018.11.102

**Published:** 2018-11-27

**Authors:** Antistio Alviz-Amador, Rodrigo Galindo-Murillo, Rafael Pineda-Alemán, Humberto Pérez-González, Erika Rodríguez-Cavallo, Ricardo Vivas-Reyes, Darío Méndez-Cuadro

**Affiliations:** a Analytical Chemistry and Biomedicine Group, Pharmaceutical Sciences Faculty, University of Cartagena, Cartagena, Colombia; b Department of Medicinal Chemistry, University of Utah, Salt Lake, USA; c Analytical Chemistry and Biomedicine Group, Exact and Natural Sciences Faculty, University of Cartagena, Cartagena, Colombia; d Department of Mathematical, Exact and Natural Sciences Faculty, University of Cartagena, Cartagena, Colombia; e Grupo de Química Cuántica y Teórica, Facultad de Ciencias Exactas y Naturales, Universidad de Cartagena, Cartagena, Colombia

**Keywords:** AMBER, Gaff, Force field parameterization, Mechanical quantic, Molecular dynamics, Geometry optimization, Validation

## Abstract

The data described here support the research article “4-HNE carbonylation induces local conformational changes on bovine serum albumin and thioredoxin. A molecular dynamics study” (Alviz-Amador et al., 2018) . Dataset on Gaff force field parameters of AMBER is provided for assembled three non-standard amino acids resulting of the 4-HNE Michael addition, the main end product of lipids peroxidation. Data include a framework for derivation of missing bonds, angles and dihedral parameters for Cys, His, and Lys modified amino acids, alongside optimized partial charges derived with Restrained Electrostatic Potential (RESP) method and the new force field parameters obtained by quantic mechanical (QM) using HF/6-31G** level of theory. Benchmark as a graphics tutorial summary steps to obtained new parameters and the validation of non-standard amino acids is presented. The new residues constructed are put available to the scientific community to perform molecular dynamics simulations of modified 4-HNE proteins.

**Specifications table**

**Table:** 

Subject area	*Biochemistry, Biophysics*
More specific subject area	*Computational Biochemistry, Computational Biophysics*
Type of data	*Figures and tables*
How data were acquired	*Quantum Mechanics (QM), Molecular Dynamics(MD), Software used: Gaussian 09 for QM, AMBER (pmemd) for MD*
Data format	*Raw and analyzed.*
Experimental factors	(2S, 4S, 5R)-4-Hydroxy-2-nonenal isomer was used to build Michael adducts
Experimental features	*Theoritical level* HF/6-31G** for QM and Gaff2 force field and ff14SB force field for MD
Data source location	*Cartagena, Colombia, Facultad de Ciencias Farmacéuticas and Facultad de Ciencias Exactas y Naturales.*10°23′58. 75°30′09., Cl. 6 #3″N, Cartagena, Bolívar
Data accessibility	*Data are supplied with this article.* Parameter files are available http://research.bmh.manchester.ac.uk/bryce/amber/
Related research article	A. Alviz-Amador, R. Galindo-Murillo, R. Pineda-Alemán, H. Pérez-González, E. Rodríguez-Cavallo, R. Vivas-Reyes, D. Méndez-Cuadro. 4-HNE carbonylation induces local conformational changes on bovine serum albumin and thioredoxin. A molecular dynamics study, J. Mol. Graph. Model. (2018). doi:10.1016/J.JMGM.2018.11.001. [1]

**Value of the data**

• Dataset of new AMBER force field parameters are provided to perform Molecular Dynamics Simulation of 4-HNE carbonylated proteins with Michael adducts on Cys, His and Lys residues.
• A benchmark framework for constructing, parameterizing, optimizing and validating of new non-standard amino acid residues modified with 4-HNE is now available.
• Our data can be used to modify, simulate and evaluate by molecular dynamic simulation the effects of 4-HNE carbonylation over any protein system.

## 1 Data

**Fig. 1:**
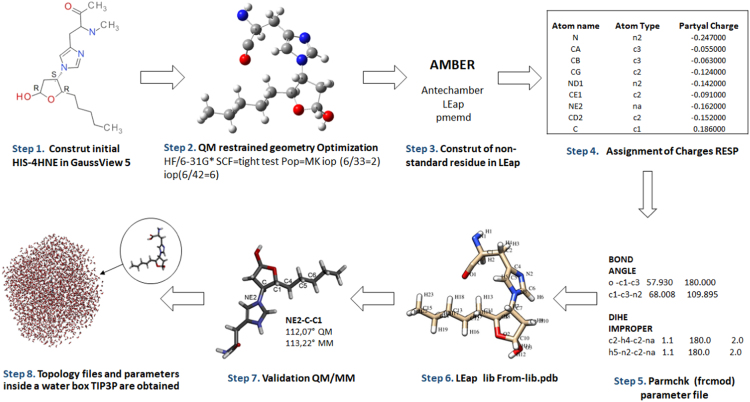
Framework for initial force field parameterization of the amino acid adduced with 4-HNE.

**Fig. 2:**
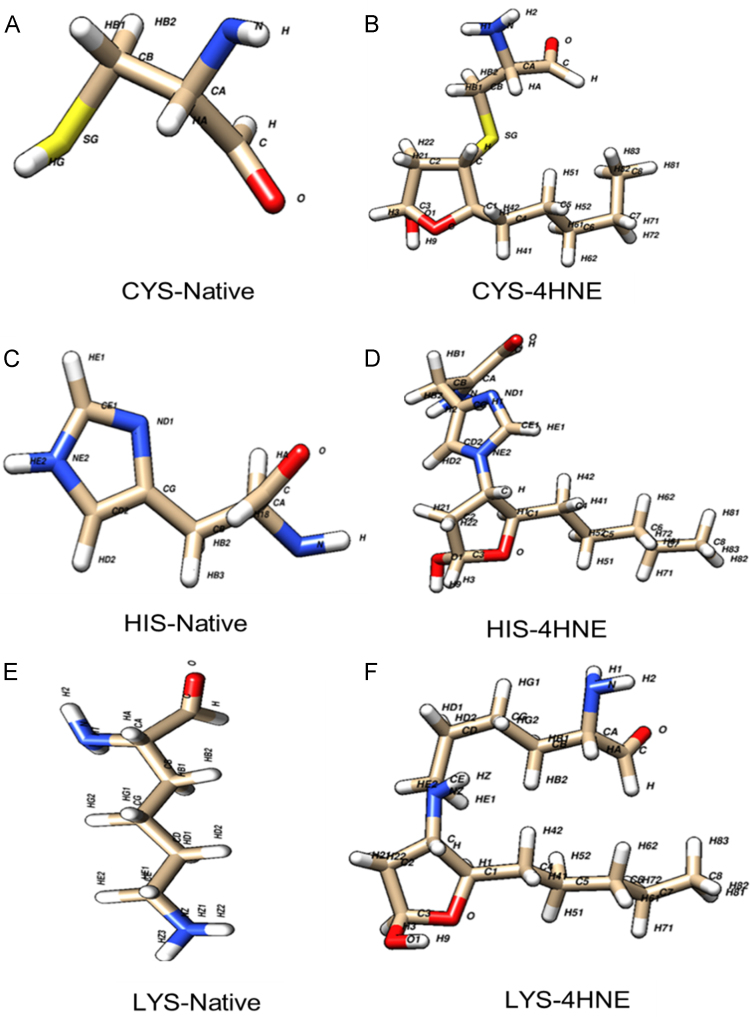
Optimized structures of non-modified and modified amino acids Cys, His and Lys with Michael adducts of 4-HNE. Figures were obtained with theory level HF/6-31G** and the atom names follows PDB conventions.

In the Supplementary material the dataset of partial charges assigned to Cys-HNE, His-HNE and Lys-HNE are shown. In Fig. 1, the workflow for preparing parameter files for non-standard residues is described. In addition, in Fig. 2 the optimized structures obtained with theory level HF/6-31G** are presented. Finally, in Tables 1–3 the dataset with the information of new obtained parameters are listed as coordinates files for Cys-HNE, His-HNE and Lys-HNE, respectively.

**Table 1:** New parameters assigned to CYS-4HNE.

***Bond***			
***Atomtypes***	***Kr***	***req***	
*c1-N*	361.821	1.431	
***Angle***			
***Atomtypes***	***KΘ***	***Θeq***	***Note***
*n2-c3-c1*	68.008	109.895	Calculated with empirical approach
*c3-c1-o*	57.930	180.000	Calculated with empirical approach
*o-c1-N*	72.926	119.420	
*c1-N-H*	50.000	120.000	
*c1-N-CX*	63.311	133.250	
*c3-c1-N*	66.420	114.600	
***Dihedral***			
***Atomtypes***	***Vn/2***	**γ (** ψ**)**	***n***
*o-c1-N-H*	1	180	2
*o-c1-N-CX*	1	180	2
*c3-c1-N-H*	1	180	2
*c3-c1-N-CX*	1	180	2

**Table 2:** New parameters assigned to HIS-4HNE.

***Bond***				
***Atomtypes***	**Kr**	**req**	**req**	
*C-n2*	361.821	1.431		
*c1-N*	361.821	1.431		
***Angle***				
***Atomtypes***	**KΘ**	**Θeq**	**Note**	
*O-C-n2*	72.926	119.420		
*C-n2-c3*	63.311	133.250		
*C-n2-hn*	46.635	118.320		
*CX-C-n2*	66.420	114.600		
*o-c1-N*	72.926	119.420		
*c1-N-CT*	50.000	121.900		
*c1-N-CX*	63.311	133.250		
*c3-c1-N*	66.420	114.600		
*n2-c3-c1*	68.008	109.895	Calculated with empirical approach	
*c3-c1-o*	57.930	180.000	Calculated with empirical approach	
***Dihedral***				
***Atomtypes***	**Vn/2**	**γ (** ψ**)**	**n**	
*O-C-n2-c3*	1	180	2	
*O-C-n2-hn*	1	180	2	
*CX-C-n2-c3*	1	180	2	
*CX-C-n2-hn*	1	180	2	
*o-c1-N-CT*	1	180	2	
*o-c1-N-CX*	1	180	2	
*c3-c1-N-CT*	1	180	2	
*c3-c1-N-CX*	1	180	2	
***Improper***				
***Atomtypes***	**Vn/2**	**γ (**ψ**)**	**n**	**Note**
*h5-n2-c2-na*	1.1	180.0	2.0	Using default value
*c2-h4-c2-na*	1.1	180.0	2.0	Using default value

**Table 3:** New parameters assigned to LYS-4HNE.

***Bond***			
***Atomtypes***	**Kr**	**Req**	**req**
*C-n3*	361.821	1.431	
***Angle***			
***Atomtypes***	**KΘ**	**Θeq**	**Note**
*O-C-n3*	72.926	119.420	
*C-n3-c3*	63.311	133.250	
*C-n3-hn*	46.635	118.320	
*CX-C-n3*	66.420	114.600	
***Dihedral***			
***Atomtypes***	**Vn/2**	**γ (** ψ**)**	**N**
*O-C-n3-c3*	1	180	2
*O-C-n3-hn*	1	180	2
*CX-C-n3-c3*	1	180	2
*CX-C-n3-hn*	1	180	2

## 2 Experimental design, materials, and methods

### 2.1 Parameterization

Dataset of Gaff force field parameters were established for three non-standard amino acids (His-HNE, Lys-HNE and Cys-HNE) to be used for molecular dynamics simulations of proteins [1] The framework for derivation of missing bond, angle and dihedral parameters is presented in Fig. 1. First, non-standard amino acid were constructed with GaussView 5, followed by full geometry optimization of the new structures using the Hartree–Fock level (HF/6-31G**) [2]. Next, assignment of charges, missing bonds, angles, and dihedral angles parameters were constructed with the antechamber and leap programs as included in AmberTools [3]. Then, charges (Step 4) of the optimized structures were calculated using RESP method [4] and the partial charges assigned to individual atoms are listed in the Supplementary material. Missing bonds, angles, and dihedral parameters of each amino acids modified with 4-HNE were established by homology, matching atom types automatically from the Gaff force field and using parmchk to generate the required force constants [5]. Dataset of new parameters assigned for the new non-standard amino acids were consigned in frcmod files and they are summarized below Tables 1–3. Next, coordinate and topology files were created for each non-standard amino acid with the program leap.

These non-standard amino acids were replaced on the proteins and the lacking parameters in frcmod files corresponding to peptide bonds, angle and torsions between the non-standard amino acids and the end nitro-terminus and the end carboxyl terminus of the nearby amino acids on proteins**,** were calculated using the program parmcal of Antechamber package. These improved frcmod files were loaded into leap program from AmberTools16 to generate the libraries files (type lib files).

Finally, the optimized structures of modified and unmodified amino acids Cys, His and Lys with Michael adducts are showed in Fig. 2; whereas the new improved parameters were included into Tables 1–3. There, bond parameters values are expressed as bond constants (**kr)** in kcal mol^−1^ Å^−2^; distance at equilibrium (**req)** in *Å*; angle constant (**kθ)** in kcal mol^−1^ degree^−2^*;* angle at equilibrium(***Θeq)*** in degrees, dihedrals constants (***Vn/2)*** in kcal/mol and dihedrals constants angles (**ψ)** in degrees.

From these datasets, the topology and coordinate of modified proteins were obtained. Hence, the applicability of the newly derived MM parameter, they were subsequently employed in 1 µs MD simulations of each of the three non-natural amino acids treated following the methodology described by [1] and [6].

### 2.2 Validation

To test the generated structures from the modified amino acids we performed MD simulations as described above using only the modified structure and compared selected bond distances and angles with structures obtained from DFT level of theory m062x/631g (d) (Table 4). Overall, good agreement between the data from high-level QM calculations and the generated AMBER structures were seen. Distance average error is between the ranges of ~0.02–0.05 Ǻ whereas angle error is within ~4°. As an additional comparison, we extracted the bonds and angles information from our modified protein systems and found that average bond difference is within ~0.01–0.03 Ǻ and within ~3.7°.

**Table 4:** Comparison between selected bond distances and angles calculated from optimized nonstandard amino acids structures. Data from the single modified amino acids were extracted from a 1 µs MD simulation using the same protocols describe before, comparisons were calculated using the DFT level of theory m062x and a basis set 6–31g (d).

**Validation methods**	**CYS-4HNE**				**HIS-4HNE**				**LYS-4HNE**			
	**Bond (Å, ± Stdev)**		**Angle (°, ± Stdev)**		**Bond (Å, ± Stdev)**		**Angle (°, ± Stdev)**		**Bond (Å, ± Stdev)**		**Angle (°, ± Stdev)**	

	SG-C	C4-C5	SG-C-C1	C4-C5-C6	NE2-C	C4-C5	NE2-C-C1	C4-C5-C6	NZ-C	C4-C5	NZ-C-C1	C4-C5-C6
**QM (m062x/631g (d)**	1.82	1.52	115.25	112.37	1.45	1.53	112.07	112.8	1.45	1.52	112	112.95
**MM (AMBER) aa alone**	1.83 ± 0.05	1.56 ± 0.02	111.77 ± 4.01	112.56 ± 4.06	1.47 ± 0.03	1.54 ±; 0.01	113.22 ± 5.17	113.40 ± 0.97	1.50 ± 0.03	1.56 ± 0.03	112.98 ± 3.29	112.36 ± 3.08
**MM (AMBER) aa on protein**	1.50 ± 0.03	1.54 ± 0.01	113.23 ± 5.17	113.41 ± 0.97	1.48 ± 0.03	1.55 ± 0.03	117.14 ± 3.3	112.73 ± 3.49	1.48 ± 0.03	1.55 ± 0.03	114.29 ± 3.42	112.82 ± 3.74
**Atoms involved in selected distances and angles**												

### 2.3 Analysis of molecular dynamics trajectories of non-standard vs. standard amino acids

**Fig. 3:**
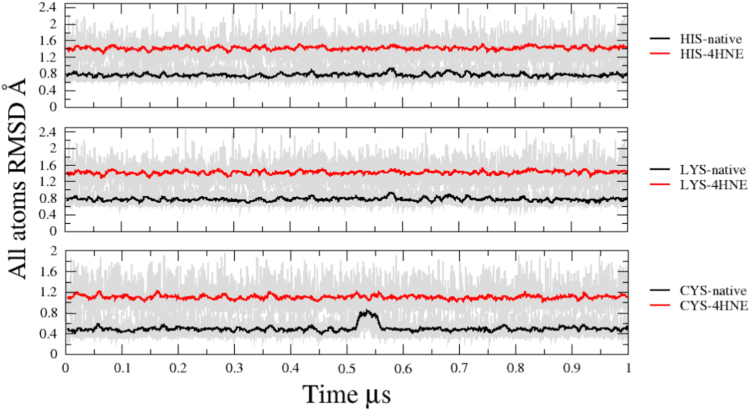
Running average (using 40 frames) of all atom RMSD of unmodified and modified amino acids with 4-HNE (non-standard) vs. time. Raw data shown in the background. A) Unmodified Histidine type HIE vs. Histidine HIE-4HNE. B) Unmodified Lysine and Lysine-4HNE. C) Unmodified cysteine vs. Cysteine-4HNE. Red line corresponding to unmodified amino acid and the black line is nonstandard amino acids. RMSD calculated using an average structure of native amino acids as a reference.

All atom root means square deviation analysis for unmodified and modified amino acids is presented in Fig. 3. Distance found in RMSD analysis for all unmodified amino acids were under 1 Å, being 0.8 Å for Hys and Lys and 0.4 Å on Cys. In the case of non-standard amino acids, RMSD values increased until an average of 1.4 Å for His-4HNE and Lys-4HNE and average of 1.2 Å for Cys-HNE (Fig. 3). Differences observed fall into a range of 0.6–0.8 Å for RSMD comparisons among modified/unmodified amino acids indicating that 4-HNE do not induce dramatically structural changes.
