# Supplementary material for: Development and benchmark to obtain AMBER parameters dataset for non-standard amino acids modified with 4-hydroxy-2-nonenal
Source: Data Brief. 2018 Nov 27;21:2581–9. doi: 10.1016/j.dib.2018.11.102 (PMC6288414; doi:10.1016/j.dib.2018.11.102)
Supplement: Supplementary file 1 — Supplementary material [file mmc1.pdf]

AUTHOR DECLARATION

We wish to confirm that there are no known conflicts of interest associated with this publication and the financial support received has not influenced its outcome..

We confirm that the manuscript has been read and approved by all named authors and that there are no other persons who satisfied the criteria for authorship but are not listed. We further confirm that the order of authors listed in the manuscript has been approved by all of us.

We confirm that we have given due consideration to the protection of intellectual property associated with this work and that there are no impediments to publication, including the timing of publication, with respect to intellectual property. In so doing we confirm that we have followed the regulations of our institutions concerning intellectual property.

We understand that the Corresponding Author is the sole contact for the Editorial process (including Editorial Manager and direct communications with the office). He/she is responsible for communicating with the other authors about progress, submissions of revisions and final approval of proofs. We confirm that we have provided a current, correct email address which is accessible by the Corresponding Author and which has been configured to accept email from dmendezc@unicartagena.edu.co

Signed by all authors as follows:

14-Nov-2018
AntistioAlviz-Amador

15-11-18
Rodrigo Galindo-Murillo

14-Nov-2018
Rafael Pineda-Alemán

Nov 14/2018
Humberto Pérez-González

14-11-2018
Ricardo Vivas-Reyes

15/11/18.
Erika Rodríguez-Cavallo

15-11-18
Darío Méndez-Cuadro
