# Supplementary material for: Development and benchmark to obtain AMBER parameters dataset for non-standard amino acids modified with 4-hydroxy-2-nonenal
Source: Data Brief. 2018 Nov 27;21:2581–9. doi: 10.1016/j.dib.2018.11.102 (PMC6288414; doi:10.1016/j.dib.2018.11.102)
Supplement: Supplementary file 2 — Supplementary material [file mmc2.docx]

**Supplementary Material**

**Data Article**

**Development and benchmark to obtain AMBER parameters dataset for non-standard amino acids modified with 4-hydroxy-2-nonenal**

**Authors**: Antistio Alviz-Amador^a^, Rodrigo Galindo-Murillo^b^, Rafael Pineda-Alemán^c^, Humberto Pérez-González, Erika Rodríguez-Cavallo^a^, Ricardo Vivas-Reyes^d^ and Darío Méndez-Cuadro*^c^

**Affiliations**:

^a.^ Analytical Chemistry and Biomedicine Group. Pharmaceutical Sciences Faculty. University of Cartagena. Cartagena-Colombia

^b^. Department of Medicinal Chemistry. University of Utah. Salt Lake. USA

^c.^ Analytical Chemistry and Biomedicine Group. Exact and Natural Sciences Faculty. University of Cartagena. Cartagena-Colombia

^d.^ Department of Mathematical. Exact and Natural Sciences Faculty. University of Cartagena. Cartagena-Colombia

^e.^ Grupo de Química Cuántica y Teórica. Facultad de Ciencias Exactas y Naturales. Universidad de Cartagena. Cartagena-Colombia

**Contact email**: [dmendezc@unicartagena.edu.co](mailto:dmendezc@unicartagena.edu.co).

Tel: + 57 3015584887

1. **Force ﬁeld Parameters for each post-translational modiﬁcation of amino acids with 4-HNE**

**Table 1. Partial charges assigned to CYS-4HNE**

| ATOM NAME | ATOM TYPE | PARTYAL CHARGE |
| --- | --- | --- |
| N  CA  C  O  CB  SG  C9  C1  C2  O2  C3  C4  C5  C6  C7  C8  O1  HA1  H3  HB1  HB2  H1  H10  H21  H22  H9  H41  H42  H51  H52  H61  H62  H71  H72  H81  H82  H83  H11 | n2  c3  c1  o  c3  ss  c3  c3  c3  os  c3  c3  c3  c3  c3  c3  oh  h1  hn  h1  h1  h1  h1  hc  hc  h2  hc  hc  hc  hc  hc  hc  hc  hc  hc  hc  hc  ho | -0.260000  -0.049000  0.214000  -0.214000  -0.275000  0.104000  -0.253000  0.037000  -0.169000  -0.310000  0.152000  -0.163000  -0.156000  -0.158000  -0.158000  -0.210000  -0.351000  0.103000  0.152000  0.133000  0.133000  0.132000  0.112000  0.119000  0.119000  0.105000  0.090500  0.090500  0.087000  0.087000  0.079500  0.079500  0.078000  0.078000  0.072000  0.072000  0.072000  0.225000 |

**Table 2. Partial charges assigned to HIS-4HNE**

| ATOM NAME | ATOM TYPE | PARTYAL CHARGE |
| --- | --- | --- |
| N  CA  CB  CG  ND1  CE1  NE2  CD2  C  O  C1  C2  C3  C4  C5  C6  C7  C8  O1  O2  C9  H2  HA  HB1  HB2  HE1  HD2  H1  H10  H31  H32  H41  H42  H51  H52  H61  H62  H71  H72  H73  H81  H82  H11  H9 | n2  c3  c3  c2  n2  c2  na  c2  c1  o  c3  c3  c3  c3  c3  c3  c3  c3  os  oh  c3  hn  h1  hc  hc  h5  h4  h1  h1  hc  hc  hc  hc  hc  hc  hc  hc  hc  hc  hc  hc  hc  ho  h2 | -0.247000  -0.055000  -0.063000  -0.124000  -0.142000  -0.091000  -0.162000  -0.152000  0.186000  -0.185000  0.009000  0.020000  -0.155000  -0.157000  -0.158000  -0.158000  -0.211000  -0.199000  -0.306000  -0.350000  0.143000  0.148000  0.100000  0.108500  0.108500  0.202000  0.180000  0.121000  0.100000  0.098000  0.098000  0.085500  0.085500  0.081000  0.081000  0.078000  0.078000  0.072333  0.072333  0.072333  0.123500  0.123500  0.223000  0.113000 |

**Table 3. Partial charges assigned to LYS-4HNE**

| ATOM NAME | ATOM TYPE | PARTYAL CHARGE |
| --- | --- | --- |
| N  CA  CB  CG  CD  CE  NZ  C  O  OXT  C9  C1  C2  O2  C3  C4  C5  C6  C7  C8  O1  H1  H2  HA  HB1  HB2  HG1  HG2  HD1  HD2  HE1  HE2  HZ  HXT  H9  H1  H21  H22  H3  H41  H42  H51  H52  H61  H62  H71  H72  H81  H82  H83  H10 | n3  c3  c3  c3  c3  c3  n3  c  o  oh  c3  c3  c3  os  c3  c3  c3  c3  c3  c3  oh  hn  hn  h1  hc  hc  hc  hc  hc  hc  h1  h1  hn  ho  h1  h1  hc  hc  h2  hc  hc  hc  hc  hc  hc  hc  hc  hc  hc  hc  ho | -0.893800  0.139500  -0.078400  -0.091400  -0.079400  0.165800  -0.820200  0.586100  -0.548000  -0.618100  0.154500  0.142100  -0.141400  -0.458600  0.330900  -0.089400  -0.078400  -0.078400  -0.080400  -0.092100  -0.630800  0.363300  0.363300  0.093700  0.061200  0.061200  0.054200  0.054200  0.049200  0.049200  0.030700  0.030700  0.376800  0.446000  0.072700  0.080700  0.070200  0.070200  0.065700  0.046200  0.046200  0.048200  0.048200  0.038200  0.038200  0.039200  0.039200  0.032033  0.032033  0.032033  0.423000 |
